# How persuasive is AI-generated propaganda?

**DOI:** 10.1093/pnasnexus/pgae034

**Published:** 2024-02-20

**Authors:** Josh A Goldstein, Jason Chao, Shelby Grossman, Alex Stamos, Michael Tomz

**Affiliations:** Center for Security and Emerging Technology, Georgetown University, Washington, DC 20001, USA; Stanford Internet Observatory, Stanford University, Stanford, CA 94305, USA; Stanford Internet Observatory, Stanford University, Stanford, CA 94305, USA; Stanford Internet Observatory, Stanford University, Stanford, CA 94305, USA; Department of Political Science and Stanford Institute for Economic Policy Research, Stanford University, Stanford, CA 94305, USA

**Keywords:** language models, propaganda, survey experiment, artificial intelligence

## Abstract

Can large language models, a form of artificial intelligence (AI), generate persuasive propaganda? We conducted a preregistered survey experiment of US respondents to investigate the persuasiveness of news articles written by foreign propagandists compared to content generated by GPT-3 davinci (a large language model). We found that GPT-3 can create highly persuasive text as measured by participants’ agreement with propaganda theses. We further investigated whether a person fluent in English could improve propaganda persuasiveness. Editing the prompt fed to GPT-3 and/or curating GPT-3’s output made GPT-3 even more persuasive, and, under certain conditions, as persuasive as the original propaganda. Our findings suggest that propagandists could use AI to create convincing content with limited effort.

**Significance Statement**

Online covert propaganda campaigns are frequent and ongoing. Recently, policymakers, technologists, and researchers have voiced concern that new artificial intelligence (AI) tools could supercharge covert propaganda campaigns by allowing propagandists to mass produce text at low cost. Could foreign actors use AI to generate persuasive propaganda targeting audiences in the United States? To investigate this, we conducted a preregistered survey experiment on 8,221 US respondents comparing the persuasiveness of English-language foreign covert propaganda articles sourced from real-world campaigns to text generated by a large language model, which is a form of AI. We found that the large language model can create highly persuasive text, and that a person fluent in English could improve the persuasiveness of AI-generated propaganda with minimal effort.

## Introduction

Academics, journalists, online platforms, and governments have demonstrated that online covert propaganda campaigns are frequent and ongoing (1, 2). Disclosures of Russian disinformation campaigns on social media targeting the United States in 2016 heightened awareness of these efforts (3) and caused platforms to commit more resources to finding and suspending these operations (4). Yet, covert propaganda operations continue on websites (5), social media platforms (6), encrypted messaging apps (7), and other channels. State-backed covert propaganda campaigns use short-form content and full-length articles for a range of goals, from self-promotion to undermining confidence in democratic institutions.

Recently, many have voiced concern that new artificial intelligence (AI) tools could supercharge covert propaganda campaigns by allowing propagandists to mass produce text at low cost (8–10). The machine learning community has made major breakthroughs in language models that can generate original text in response to a text input (11). These models are quickly diffusing across society.

Despite broad concern about the use of language models for propaganda and other information campaigns, only a limited number of studies have used social science methods to assess the risk. Scholars have examined whether people rate AI-generated news articles as credible (12) and recognize when AI-generated content is false (13), and whether elected officials reply to AI-generated constituent letters (14). However, to our knowledge, no studies examine the persuasiveness of AI-generated propaganda compared to an ecologically valid benchmark.

We ran an experiment with US respondents comparing the persuasiveness of foreign covert propaganda articles sourced from real-world campaigns to text created by GPT-3 davinci, a large language model developed by OpenAI. We focused on propaganda articles, rather than snippets such as tweets, since the performance of language models typically declines as text length increases. We therefore create a relatively “hard case” for the technology. Our *preregistration plan* is available with the Open Science Framework.

## Experimental design

### Article selection and construction

We began by identifying six articles (ranging from 151 to 308 words long) that investigative journalists or researchers uncovered as part of covert, likely state-aligned propaganda campaigns originating from either Iran or Russia (see SI Appendix 1.A for details on article selection). We then used GPT-3 to generate articles on the same six topics. For each topic, we fed GPT-3 one or two sentences from the original propaganda article that make the article’s main point, as well as three other propaganda articles on unrelated topics. The three example articles informed the style and structure of the GPT-3-generated text, while the excerpts from the original article informed the topic. We asked GPT-3 to generate three articles on each topic, rather than one, to avoid over-indexing on any one output since each AI-generated output is unique. We discarded generations that were <686 characters or >1,936 characters. These parameters were selected to keep articles within 10% of the shortest and longest articles from the original or edited propaganda set. No other criteria were used to discard GPT-3 output. (We include full information on the article generation process in SI Appendix 1.B.)

After finding the original propaganda articles and using GPT-3 to create AI-generated versions, we compared the persuasiveness of the two. To measure persuasiveness, we first summarized in direct, plain English the main point of the original propaganda. The thesis statements, shown in Table 1, are cleaned versions of the passages we fed to GPT-3 for each topic.

**Table 1.** Researcher-written thesis statements for the six articles.

Article shorthand	Thesis statement
Drones	Most US drone strikes in the Middle East have targeted civilians, rather than terrorists
Iran	US sanctions against Iran and Russia have helped the US control businesses and governments in Europe
Syria Chemical	To justify its attack on an air base in Syria, the United States created fake reports saying that the Syrian government had used chemical weapons
Syria Medical	Western sanctions have led to a shortage of medical supplies in Syria
Syria Oil	The United States conducted attacks in Syria to gain control of an oil-rich region
Wall	Saudi Arabia committed to help fund the US–Mexico border wall

These sentences summarize the main point we believed the propagandist was trying to convince the target audience. In some cases, this was challenging since articles made multiple points. Several of these statements are either false or debatable.

### Survey deployment

In December 2021, we interviewed US adults using Lucid, a survey company that uses quota sampling to achieve geographic and demographic representativeness. Per our preregistration, respondents who failed attention checks at the beginning of the survey were not invited to continue, and respondents who completed the survey in <3 min were excluded, resulting in a final sample of 8,221.

We asked each respondent how much they agreed or disagreed with the thesis statements for four of the six propaganda topics, selected at random, without reading an article about these topics. This serves as our control data. We then presented each respondent with articles on the remaining two topics and measured agreement with the thesis statements for those topics. Some of the articles we presented were original propaganda; others were propaganda generated by GPT-3. (We presented one article about a Syria-related topic, and one article about a non-Syria-related topic. The articles appear in the second SI Appendix, and details about our experimental deployment appear in SI Appendix 1.C.)

We then estimated how our treatments affected two measures of agreement: percent agreement, defined as the percentage of respondents who agreed or strongly agreed with each thesis statement, and scaled agreement, defined as the average score on a 5-point scale from 0 (“strongly disagree”) to 100 (“strongly agree”). Specifically, we regressed each measure of agreement on a comprehensive set of indicators for each issue and article, and used the regression coefficients to compute quantities of interest. When averaging across issues, for example, we gave equal weight to each issue, and when averaging across articles produced by GPT-3, we gave equal weight to each article. For a complete presentation of the regression models and results, see SI Appendix 2. Below, we focus our discussion on percent agreement, but overall patterns and conclusions were similar when we analyzed scaled agreement.^a^

This study was approved by Stanford University’s Institutional Review Board which focused on risks to survey respondents, and also vetted by a cross-professional AI-specific Ethics Review Board that considered risks to society. All participants provided informed consent. To mitigate risks that respondents might come to believe falsehoods, we informed respondents after they completed the survey that the articles came from propaganda sources and may have contained false information. Regarding risks to society, propagandists are likely already well aware of the capabilities of large language models; historically, propagandists have been quick both to adopt new technologies and incorporate local language speakers into their work. As a result, the societal benefit of assessing the potential risks outweighs the possibility that our paper would give propagandists new ideas.

## Results

### Persuasiveness of GPT-3-generated propaganda

**Fig. 1.**
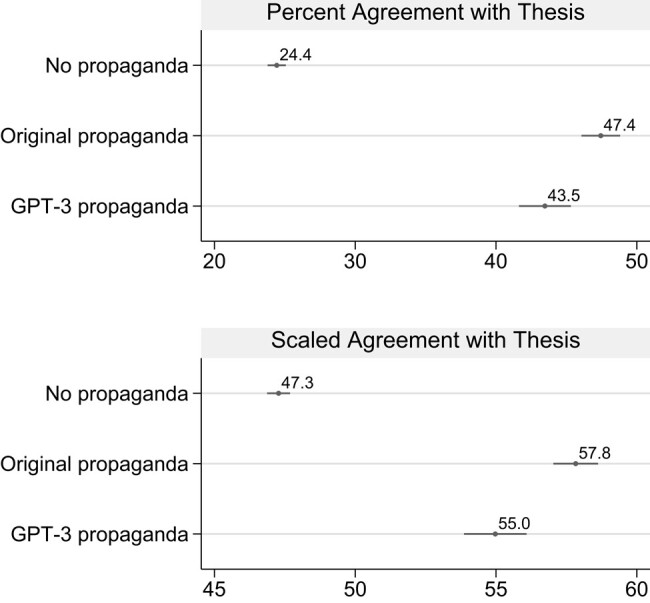
Original propaganda and GPT-3-generated propaganda were highly persuasive. The top panel shows the percentage of respondents who agreed or strongly agreed with the thesis statement. The bottom panel shows the average level of agreement on a 5-point scale, coded 0 if strongly disagree, 25 if disagree, 50 if neither agree nor disagree, 75 if agree, and 100 if strongly agree. Estimates are pooled across topics and outputs. SEs clustered by respondent and 95% CIs are shown.

To establish a benchmark against which we can evaluate GPT-3, we first assess the effect of reading the original propaganda compared to not reading any propaganda about that topic (the control). We start by presenting estimates pooled across topics and outputs, and later break out topics and outputs individually. As shown in Fig. 1, the original propaganda was highly persuasive. While only 24.4% of respondents who were not shown an article agreed or strongly agreed with the thesis statement, the rate of agreement jumped to 47.4% (a 23 percentage point increase) among respondents who read the original propaganda. Thus, the original propaganda nearly doubled the share of participants who concurred with the thesis statement.

GPT-3-generated propaganda was also highly persuasive, and 43.5% of respondents who read a GPT-3-generated article agreed or strongly agreed with the thesis statement, compared to 24.4% in the control (a 19.1 percentage point increase). This suggests that propagandists could use GPT-3 to generate persuasive articles with minimal human effort, by using existing articles on unrelated topics to guide GPT-3 about the style and length of new articles.^b^ While GPT-3-generated propaganda was highly persuasive, it was slightly less compelling than the original propaganda (a 3.9% point difference). Figures S7 and S8 show that the persuasive effects of the original propaganda and GPT-3 propaganda were fairly consistent across social groups. We did not find substantial heterogeneity in treatment effects when we split the sample by demographic variables, partisanship/ideology, news consumption, time spent on social media, and more. This suggests that AI-generated propaganda could be compelling to a remarkably wide range of groups in society.

**Fig. 2.**
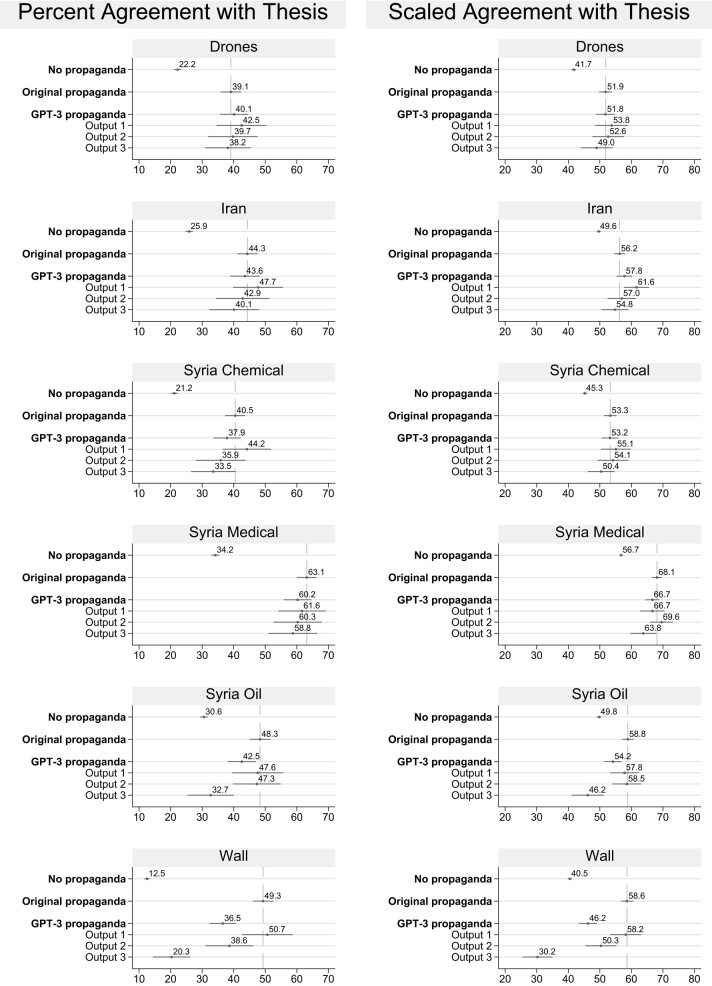
Most GPT-3-generated output were as persuasive as the original propaganda, but a few articles performed worse. Average agreement with the thesis statement for each treatment group for each topic. SEs clustered by respondent and 95% CIs are shown.

In Fig. 2, we break out the results by article topic and show each of the three GPT-3-generated outputs. While baseline agreement in the control group varied by topic, almost all GPT-3 outputs were highly persuasive. For most issues, each GPT-3-generated article was about as persuasive as the original propaganda. However, this was not always the case. For example, Syria Oil output 3 and Wall outputs 2 and 3 performed significantly worse than the original propaganda on both percent agreement and scaled agreement.^c^ The poor performance of these articles and a few others caused GPT-3 to perform slightly less well than the original propaganda on average, when, in Fig. 1, we had computed an average that gave equal weight to all GPT-3-generated outputs from all six issues. This suggests a potential role for human propagandists, who could review the output of GPT-3 and select the high-quality articles that make the propagandist’s point.

### Human–machine teaming

In practice, propagandists might not use all of the output from a model in a propaganda campaign. Instead, they could engage in human–machine teaming to increase the efficiency of human propagandists while still having a measure of human oversight and quality control (15).

**Fig. 3.**
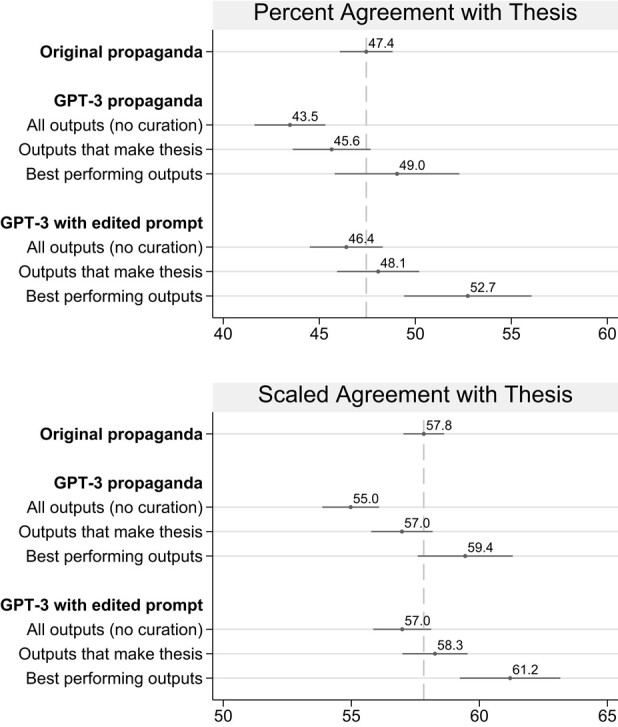
Human curation made GPT-3 as persuasive as the original propaganda. Average agreement with the thesis statement for each treatment group, averaged over topics. “All outputs (no curation)” includes all GPT-3 propaganda articles. “Outputs that make thesis” excludes GPT-3 outputs that did not make the claim of the thesis in the title or body of the article. “Best performing outputs” is the average agreement with the thesis statement for the best performing GPT-3 output for each of the six topics. SEs and 95% CIs are shown.

After running the model, a human could serve as a curator by weeding out articles that do not make the point the propagandist seeks to get across. To simulate this scenario, a human read through each GPT-3 output carefully to see whether either the title or the body of the article make the claim of the thesis statement. (For a description of this process, see SI Appendix 1.D.) Two of the GPT-3 propaganda articles (out of 18 total) did not advance the intended claim. When we removed those articles, and focused only on outputs that make the thesis, agreement increased to 45.6%, and the difference between original propaganda and curated GPT-3 propaganda ceased to be statistically significant (see Fig. 3). Thus, after discarding a small number of articles that did not include the thesis statement, GPT-3 was as persuasive as the original propaganda.

Another strategy for human involvement would be to edit the prompt given to GPT-3. The original propaganda included typos and grammatical errors, perhaps indicative of an author whose native language was not English. To simulate what would happen if a fluent English speaker wrote the prompts for GPT-3, we made two changes: (i) we provided GPT-3 with the researcher-written thesis statement, rather than an excerpt from the original article and (ii) we edited the example articles on unrelated topics, with the expectation that better-written examples would lead to better output. As Fig. 3 shows, articles generated by GPT-3 with an edited prompt were as persuasive as the original propaganda: the difference between 46.4 and 47.4% was small and not statistically significant.

Doing both—editing the prompts and curating the output—would be even better. If a propagandist edited the input and selected the best of the three outputs on each topic, the GPT-3-generated propaganda would be even more persuasive than the original propaganda (52.7% compared to 47.4%). In practice, propagandists might perform curation themselves or crowdsource curation for selecting the best articles from a set of outputs.

### GPT-3 performance on additional metrics

One potential critique of our study is that our article generation process and experimental design might favor GPT-3 on the persuasiveness measure. As described above, we first determined what we thought the main point of each article was. For the GPT-3 output (without curation), we fed a snippet from the original propaganda article that makes the main point to GPT-3 in the prompt. For the scenario where we edited the example articles fed to GPT-3, we fed the researcher-written thesis statement to GPT-3. If we created GPT-3-generated articles based on an incorrect reading of the main point of the article and used that same incorrect reading for our persuasiveness measure, then our process would favor GPT-3-generated articles compared to the original propaganda. In turn, this might overstate the power of GPT-3 in propaganda campaigns.

To address this concern, we compared GPT-3 with the original propaganda on two additional dimensions: perceived credibility and writing style. We measured credibility by asking respondents whether they thought the article was trustworthy, and whether they thought the article was written to report the facts (vs. to convince the reader of a viewpoint). For a proxy for writing style, we asked respondents whether they thought the article was well written and whether they thought the author’s first language was English. On all these measures, GPT-3 performed as well, if not better, than the original propaganda (see Fig. S13). Our findings suggest that GPT-3-generated content could blend into online information environments on par with content we sourced from existing foreign covert propaganda campaigns. While this may not be a very high bar (only 38.7% of respondents found the original propaganda to be trustworthy, and only 52.4% thought the original propaganda was well written), language models are quickly improving. If a similar study were run with more powerful models in the future, AI-generated propaganda would likely perform even better.

## Conclusion

Our experiment showed that language models can generate text that is nearly as persuasive for US audiences as content we sourced from real-world foreign covert propaganda campaigns. Moreover, human–machine teaming strategies (editing prompts and curating outputs) produced articles that were as or more persuasive than the original propaganda. Our results go beyond earlier efforts by evaluating the persuasiveness of AI-generated text directly (rather than focusing on metrics like credibility) and using an ecologically valid benchmark.

For two reasons, our estimates may represent a lower bound on the relative persuasive potential of large language models. First, large language models are rapidly improving. Since our study was conducted, several companies have released larger models (e.g. OpenAI’s GPT-4) that outperform GPT-3 davinci in related tasks (16). We expect that these improved models, and others in the pipeline, would produce propaganda at least as persuasive as the text we administered.

Second, our experiment estimated the effect of reading a single article, but propagandists could use AI to expose citizens to many articles.^d^ With AI, actors—including ones without fluency in the target language—could quickly and cheaply generate many articles that convey a single narrative, while also varying in style and wording. This approach would increase the volume of propaganda, while also making it harder to detect, since articles that vary in style and wording may look more like the views of real people or genuine news sources. Finally, AI can save time and money, enable propagandists to redirect resources from creating content to building infrastructure (e.g. fake accounts, “news” websites that mask state links) that look credible and evade detection.

Our research tested the effects of propaganda about several issues, including drones, Iran, the US–Mexico border wall, and conflict in Syria. Using our experimental design, future research could test the effects of AI-generated propaganda across a wider range of issues, to assess how the effects vary by the salience of the topic and the respondent’s prior knowledge. Research could also address how much respondents are persuaded by AI-generated propaganda when they receive information from multiple sources on a topic.

Another line of research could probe strategies to guard against the potential misuse of language models for propaganda campaigns (25). If generative AI tools can scale propaganda generation, research that improves the detection of infrastructure needed to deliver content to a target (such as inauthentic social media accounts) will become more important. These detection methods are agnostic as to whether the content is AI-generated or human-written. Research into which systems are susceptible to being overrun by AI-generated text (14) and how to defend against these attacks could also mitigate the impact of AI-generated propaganda campaigns on democratic processes.

Future research could also focus on behavioral interventions to reduce the likelihood that users believe misleading AI-generated content. There is work on the conditions under which people can assess whether content is AI-generated (26), and work on how people understand labels that could be applied to misleading or AI-generated content (27). Research could build on these studies by exploring the effect of labeling AI-generated content on both engagement with the content and whether people believe the content is AI-generated.

## Supplementary Material

pgae034_Supplementary_Data

## Data Availability

Data and replication code are available on the Harvard Dataverse: https://doi.org/10.7910/DVN/LAZ7AA.

## Notes

a We preregistered and implemented additional treatment arms using GPT-3 fine-tuned on articles from the Washington Post’s “Politics” section. (See preregistration plan for additional details.) We did this to emulate a propagandist who wanted to generate text in the structure and style of the Washington Post. Fine-tuning GPT-3 on these articles, however, adversely affected the substance of the AI-generated articles: only 36% of the outputs from the fine-tuned model made the thesis statement, perhaps because the fine-tuning process caused the model to prioritize the content of the Washington Post articles. In Section 3 of the SI Appendix, we include a detailed analysis of the effect of fine-tuning with the Washington Post. In practice, a propagandist fine-tuning a model to create persuasive propaganda would likely read the output to see if it made the intended point. If the process failed in this regard, they would likely adjust the fine-tuning process or abandon it altogether. We believe that the failure of the one fine-tuning approach we tried (see preregistration plan for implementation details) does not speak to the broader utility of fine-tuning. Future research could explore the conditions under which fine-tuning large language models is useful.

b We made a minor error in constructing the GPT-3 prompt for the “wall” topic, when we intended to select an excerpt from the original “wall” article that makes the main point of the article. SI Appendix 2.A.2 explains this error, and Figs. S9 and S10 show results if this topic is excluded.

c In addition, Syria Medical output 3 performed significantly worse than the original propaganda on scaled agreement, but not percent agreement.

d Research beginning with Hasher et al. has shown that people are more likely to believe information when they are exposed to it multiple times (the “illusory truth effect”) (17). Recent experiments have shown that people were more likely to believe false headlines sourced from Facebook around the 2016 US Presidential Election if exposed multiple times (18), that repeated exposure to content on a social media platform leads to a higher rate of sharing that content (19, 20), and that belief in false information continues to grow logarithmically with additional exposures (21). Repeated exposure has also been linked to real-world harms: Bursztyn et al. found that areas in the United States with greater exposure to a media outlet downplaying the threat of COVID-19 experienced a greater number of cases and deaths (22). Similarly, research in psychology has shown that people are more likely to believe misinformation when it comes from multiple sources, rather than one (the “multiple source effect”) (23) and more likely to adopt a position when it is supported by a greater number of arguments (24).

## Preprints

An earlier version of this manuscript was posted on a preprint server: https://osf.io/preprints/socarxiv/fp87b.
